# Anti-TB Drugs for Drug-Sensitive and Drug-Resistant *Mycobacterium tuberculosis*: A Review

**DOI:** 10.3390/cimb47090776

**Published:** 2025-09-19

**Authors:** Kara Lukas, Madeleine T. Dang, Clare Necas, Vishwanath Venketaraman

**Affiliations:** College of Osteopathic Medicine of the Pacific, Western University of Health Sciences, Pomona, CA 91766, USA; kara.lukas@westernu.edu (K.L.); madeleine.dang@westernu.edu (M.T.D.);

**Keywords:** tuberculosis, drug-resistant, drug-sensitive, metformin, CRISPR, N-acetylcysteine, glutathione, *Mycobacterium tuberculosis*

## Abstract

Tuberculosis (TB) is a global health challenge caused by *Mycobacterium tuberculosis,* with drug resistance, treatment toxicity, and treatment adherence challenges continuing to impede control efforts. The objective of this review is to explore current advancements in TB treatment, for both drug-sensitive and drug-resistant TB, focusing on pharmacologic regimens, diagnostics, and adjunctive therapies. For drug-sensitive TB, a 4-month rifapentine–moxifloxacin regimen has been proven to be non-inferior to the traditional 6-month standard, while optimized pyrazinamide dosing or faropenem substitution may improve culture conversion and reduce adverse events. In drug-resistant TB, regimens such as the bedaquiline, pretomanid, linezolid, and moxifloxacin have demonstrated efficacy with substantially shorter treatment duration; however, incidents of hepatotoxicity and linezolid-related neuropathy require careful monitoring. Adjunctive therapies, such as metformin, N-Acetylcysteine, aspirin, and statins, show promising effects in modulating host immunity and reducing long-term lung damage. Advances in diagnostics, including whole genome sequencing and CRISPR-based methods, are enabling rapid detection of resistance mutations and directed therapy. Vaccine development has advanced beyond the BCG vaccine to explore vaccines with enhanced immunogenicity or ones that are safe for immunocompromised patients. Implementation strategies such as video directly observed therapy are improving adherence; additionally, community-based, technology-supported interventions significantly improve TB knowledge and compliance. An integrated approach that combines optimized pharmacologic regimens, host-directed therapies, advanced diagnostics, and patient-centered public health strategies is essential to reduce TB incidence, long-term morbidity, and mortality.

## 1. Introduction

Tuberculosis (TB) is one of the world’s most critical health challenges despite being preventable and typically curable [1,2]. According to the 2024 World Health Organization (WHO) report, in 2023 TB became the world’s leading cause of death from a single infectious agent after having been replaced for three years by coronavirus disease [2]. As a highly contagious airborne disease, TB is caused by *Mycobacterium tuberculosis* and most commonly affects the lungs, and is thus referred to as pulmonary TB [1,3]. It is estimated that a quarter of the world’s population carries a latent TB infection, which can progress to become an active disease, especially in individuals with compromised immune systems [1,4].

**Figure 1:**
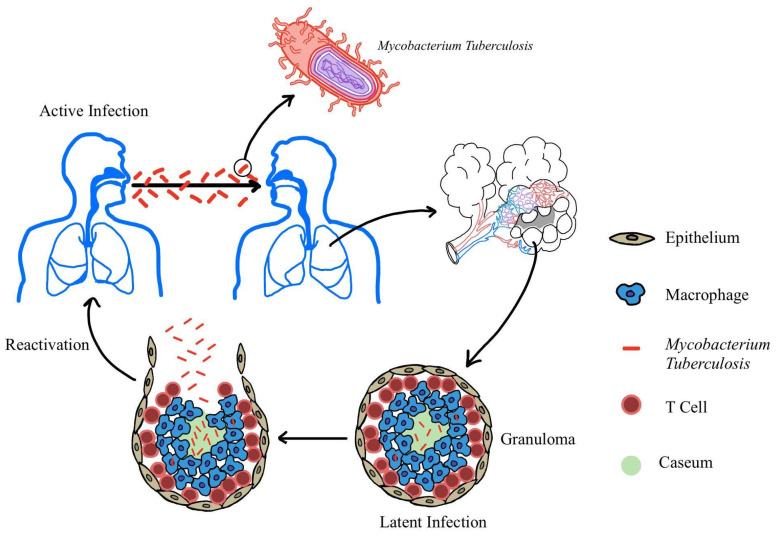
Overview of TB Infection *. * Imaging of the alveoli and granuloma adapted from Rahlwes et al. [6] and Sarathy et al. [11], respectively. *M. tuberculosis* has a thick, complex cell wall that is transmitted via inhaled aerosols and phagocytosed by alveolar macrophages that eventually form granulomas. Once the granuloma forms, it can enter a latent stage in immune competent individuals. However, in immunocompromised individuals, the granuloma can become reactivated and burst open, releasing *M. tuberculosis* and causing an active TB infection.

As seen in Figure 1, *M. tuberculosis* is transmitted via inhaled aerosols and is phagocytosed by alveolar macrophages, triggering a proinflammatory response that leads to granuloma formation [5,6]. In people with competent immune systems, the body forms a robust granuloma composed of various cells, including neutrophils, dendritic cells, T cells, and fibroblasts. The granuloma contains *M. tuberculosis* bacilli, leading to latent TB [7]. However, in immunocompromised individuals, *M. tuberculosis* can lead to active TB or can “reactivate” the latent TB infection, which then is able to spread to other parts of the body [6]. *M. tuberculosis* has a thick, complex cell wall that slows its growth and contributes to drug resistance. It resists treatment by modifying drug targets, inactivating drugs, and using efflux pumps [8]. It also produces virulence factors, such as early secreted antigenic target 6 (ESAT-6), which disrupts immune defenses and promotes bacterial spread by damaging macrophages and rupturing phagosomes [4,9,10].

In drug-sensitive TB, the current recommended first-line treatment includes isoniazid (INH), rifampicin (RIF), pyrazinamide (PZA) and ethambutol (EMB) [2,12]. However, the emergence of drug-resistant TB poses a major global health challenge, as mutations in *M. tuberculosis* can reduce the efficacy of standard first-line therapies, leading to prolonged infection, treatment failure and increased risk of further resistance [8]. The current recommended first-line treatment for drug-resistant TB includes bedaquiline, pretomanid, linezolid, and moxifloxacin (BPaLM) [2]. Drug toxicity in the treatment of both drug-sensitive and drug-resistant TB includes hepatotoxicity, thrombocytopenia, and leukopenia. These toxicities present a limitation in the current TB treatment regimens and often necessitate deviations from standard treatment [9]. Significant adverse drug reactions, such as loss of visual acuity, fever, itching, arthralgia, fatigue, and paresthesia of the tongue, causes 9.5% of patients to change their treatment regimens [13]. Cumulative toxicity from prolonged multidrug therapy highlights the importance of developing shorter, safer regimens to minimize long-term complications [8]. Shorter regimens and alternative therapies are thus being explored to improve treatment adherence, reduce toxicity, and address emerging challenges in TB management, particularly in populations with comorbidities or at risk of poor outcomes [14,15,16].

The objective of this review is to explore, summarize, and critically evaluate recent advancements in the treatment and management of both drug-sensitive and drug-resistant TB, with an emphasis on pharmacologic regimens, diagnostics, and adjunctive therapies.

## 2. Methods

We conducted a literature search in PubMed from June 2025 through September 8th, 2025, using the terms “Anti-TB drugs for drug sensitive and drug resistance Mycobacterium tuberculosis”, “Anti-tuberculosis drugs”, and “mycobacterium tuberculosis” in combination with additional keywords such as “rifampicin”, “antibiotics”, “glutathione”, “metformin”, “diabetes”, “drug sensitive”, “drug resistance”, “CRISPR”, “vaccines”, “epidemiology”, and “adjunctive treatment.”

Articles were screened based on title and abstract, followed by full-text review. We included studies primarily published within the last five years that were relevant to TB pharmacological therapy or adjunctive treatment strategies. Priority was given to randomized controlled trials, meta-analyses, systematic reviews, and clinical trials. We excluded studies that were not directly related to TB treatment or drug development.

## 3. Diagnosing TB

Multi-drug-resistant TB (MDR-TB) is diagnosed using drug susceptibility tests (DST), such as nitrate reductase assay, Roche solid ration method, liquid culture method, and many genomic testing methods. Within the current DST methods, there are limitations. The most prevalent limitations are the time and resources it takes to complete a test; for example, one test can take 28–42 days to complete [17,18,19,20].

With the continuous advancement of various molecular techniques, such as Whole Genome Sequencing (WGS), patients with drug-resistant TB can be detected earlier [21]. Feng et al. [22] noted that TB mutations spontaneously arise at low frequency (2 × 10^−10^) [22]. The WHO published an updated TB mutation catalog in 2023, which has allowed researchers to use WGS and analysis tools to better predict and monitor TB resistance [21,23]. In China, a high TB burdened country, a first edition WHO catalog is used to see how mutations can differ by country. Pei et al. [24] found a mutation within their population that was not present in either the first or second edition WHO catalog, which demonstrates the evolving nature of TB and the importance of continued research [24].

With antibiotic resistance continuing to persist among new cases and those not responding to the initial treatment, novel DSTs are needed to detect new mutations within populations and develop the necessary drugs to treat them [2]. Clustered Regularly Interspaced Short Palindromic Repeats-CRISPR associated (CRISPR-CAS) has been used in TB research to find possible mutations at an accelerated rate [22], to develop new assays [25], and to make resistant mycobacteria susceptible to antibiotics again [26]. Feng et al. [22] created a CRISPR-guided DNA polymerase system named Cas9 and Mutagenic Polymerase for Evolving Resistance (CAMPER) to increase the mutation frequency in TB [22]. When CAMPER was tested on the RIF resistance-determining region (RRDR) of the *rpoB* gene, thirty-one single guide RNA were designed and five regions showed higher mutation frequencies, ranging from 5-fold to 97-fold than the off-target control. This enabled CAMPER to be used to rapidly discover drug-resistant mutations [22]. Another study investigated the efficacy of a Cas9/gRNA-assisted quantitative Real-Time PCR (CARP) assay to detect point mutations in the *rpoB* gene of TB. The CARP assay showed a high specificity in detecting the two point mutations that make up over 60% of the RIF-resistant cases. This finding raises the possibility of extending the application to other mutations as well as to the more severe forms of drug-resistant TB, total drug resistant and extreme drug-resistant TB [25]. Additionally, Sodani et al. [26] studied the use of CRISPR-Cas9 on *Mycobacterium smegmatis* (*M. smegmatis*), a non-pathogenic species that is closely related to *M. tuberculosis* [26,27]. Investigators found that introducing double stranded breaks proved to be fatal to the bacteria. Sodani et al. [26] also resensitized hygromycin-resistant *M. smegmatis* by targeting a plasmid that conferred hygromycin resistance. This demonstrated how CRISPR-Cas has the potential to selectively target cells with mutations [26]. These studies show the importance of continued TB research to rapidly detect and treat these mutations.

## 4. Vaccines

For TB, the only approved vaccine, the *M. bovis Bacille-Calmette-Guérin* (BCG), is given to infants intradermally [28]. While the BCG vaccine is shown to be significantly protective in children under the age of five, it becomes less protective by 12.5 years [29]. With this knowledge, novel vaccines are being created, including ones that enhance the effectiveness of the current BCG vaccine, that replace the current BCG vaccine, and that are effective at various stages of TB manifestation [28].

Preclinical and clinical trials are primarily focusing on recombinant BCG, subunit vaccines, attenuated live vaccines, mRNA vaccines, viral vector vaccines, and inactivated vaccines as well as alternative routes of administration [30,31]. One study that looked at mice that were intranasally administered NanoSTING-H1, a liposomal nanoparticle, found that there was comparable protection to the BCG vaccine when challenged with TB strain Erdman. This study provides an alternative to the BCG vaccine, especially for those that are immunocompromised, because it is not a live attenuated vaccine like the current BCG vaccine [31]. Another study looked at a subunit vaccine, H107, which was designed to complement the current BCG vaccine by containing eight specific protective antigens against TB (EsxA, EspA, EspC, EspI, MPT64, MPT70, MPT83, and PPE68) [32,33]. H107 is particularly important because coadministration with the BCG vaccine produced lower differentiated CD4 Th1 cells and a higher Th17 immune response, increasing vaccine efficacy [33]. When comparing the BCG vaccine in mouse models to recombinant BCG, rBCG-ECD003 increased levels of CD4+ T-cells, produced long-lasting humoral activity, and enhanced immunogenicity [34]. For clinical trials, one particular attenuated live vaccine, MTBVAC, is important because it is being considered as a booster vaccine for adolescents and adults, in addition to being a new vaccine for neonates [35]. The clinical trials NCT02933281, NCT03536117, and NCT04975178, a Phase 1a/2b, Phase 2a, and Phase 3 trial, respectively, are exploring the safety and immunogenicity of this vaccine [30]. While this is not an extensive list of all the preclinical and clinical trials being conducted, the aforementioned trials provide insight into the importance of creating a novel vaccine to combat the TB epidemic, one of the key commitments that the WHO listed in their 2024 Global Tuberculosis Report [2].

## 5. Anti-TB Drugs

### 5.1. Drug-Sensitive TB

Current treatment strategies for drug-susceptible TB are undergoing significant research to improve efficacy, shorten treatment duration, and minimize adverse effects. The American Thoracic Society and the Infectious Disease Society of America updated their treatment guidelines in 2024 to support a 4-month regimen consisting of INH, rifapentine, moxifloxacin and PZA for patients with INH-susceptible, RIF-susceptible TB [36]. Several phase 3 randomized controlled trials have demonstrated the non-inferiority of 4-month rifapentine–moxifloxacin-based regimens compared to the previously recommended 6-month regimen [14,37]. Chang et al. [37] showed that high-dose rifapentine combined with moxifloxacin was non-inferior to the standard 6-month treatment; regimens replacing moxifloxacin with EMB failed to meet the non-inferiority criteria [37]. Additionally, Dorman et al. [14] confirmed the comparable cure rate of a rifapentine–moxifloxacin regimen across multiple populations. However, the rifapentine-only regimen did not meet non-inferiority criteria, indicating the importance of moxifloxacin use in regimen shortening [14].

In addition to shortening the duration of treatment, there is continuing research into the optimization of the pharmacokinetic and safety profiles of the currently recommended anti-TB drugs. A pooled analysis of three phase 2 clinical trials suggests that higher concentrations of PZA for 12 weeks is associated with higher culture conversion rates in the treatment of drug-susceptible TB. Based on the modeling, the optimal dose for PZA likely is between 35 and 45 mg/kg [38]. However, recent findings from Xu et al. [39] challenged the traditional weight-based dosing approach. Their analysis found that flat dosing PZA at 1000 mg for 4 months led to the greatest proportion of participants achieving therapeutic concentrations compared to weight-based dosing and flat dosing at 1500 mg. Dose-dependent clearance of PZA highlights that standard flat dosing may offer more consistent drug exposure across patient populations [39]. Additionally, Choi et al. [13] found that the incidence of hepatobiliary adverse reactions peaked during the intensive phase; they suggest that PZA administration may be a causal factor of hepatotoxicity [13].

Drug substitutions are also being explored to improve tolerability [40,41]. A 2023 randomized controlled trial found that substituting faropenem for EMB in the standard 6-month TB treatment regimen was non-inferior to the standard treatment, while also avoiding EMB-related ocular toxicity and reducing overall side effects [36]. Novel agents are likewise being evaluated. Dooley et al. [41] found that regimens including 12 weeks of pretomanid alongside rifamycin and PZA exhibited higher microbiological activity than standard therapy; however, there was an increased risk of adverse events and a greater number of asymptomatic liver transaminase elevations [41].

A recent phase 2 early bactericidal activity (EBA) study evaluated sitafloxacin in patients with drug-susceptible TB to compare its performance to levofloxacin and INH over seven days of monotherapy. Sitafloxacin, which has been demonstrated to have bactericidal effects against drug-resistant strains, showed similar EBA and greater prolonged EBA compared to levofloxacin and INH. These findings suggest that sitafloxacin may be a promising option for both drug-susceptible and drug-resistant TB regimens [42].

As seen in Table 1, these studies highlight the dynamic landscape of research for anti-TB drug development and optimizations with hopes of shorter, more tolerable, and more effective treatments.

**Table 1:** Summary of novel and alternative drug-sensitive TB treatment regimens compared to the standard WHO-recommended 6-month regimen. Regimens include shortened durations, modified dosing strategies, or non-standard drugs currently under investigation in clinical trials or recent studies. Key findings include the primary efficacy outcomes reported in each study relative to the standard regimen.

Regimen	Drugs Used	Treatment Duration	Key Findings	Study/Source
Standard Treatment (Reference)	Isoniazid, Rifampicin, Pyrazinamide, Ethambutol	6 months	Baseline Comparison Regimen	[2]
4-month Rifapentine–Moxifloxacin Regimen	Isoniazid, Rifapentine, Moxifloxacin, Pyrazinamide	4 months	Non-inferior to 6-month standard regimen; moxifloxacin essential for efficacy	[14,36,37]
Higher Dose Pyrazinamide Regimen	Standard drugs with Pyrazinamide 35–45 mg/kg	12 weeks	Higher culture conversion rates	[38]
Flat-Dosing Pyrazinamide Regimen	Standard drugs with Pyrazinamide 1000 mg	4 months	1000 mg flat dose achieves most consistent therapeutic levels	[39]
Faropenem Substitution Regimen	Isoniazid, Rifampicin, Pyrazinamide, Faropenem (instead of Ethambutol)	6 months	Non-inferior to standard treatment; fewer side effects; avoids ocular toxicity	[40]
Pretomanid-Containing Regimen	Pretomanid, Rifamycin, Pyrazinamide	12 weeks	Higher microbiological activity; increased risk of adverse events	[41]
Sitafloxacin Monotherapy Regimen	Sitafloxacin Monotherapy	7 days	Similar EBA and compared to levofloxacin and isoniazid greater prolonged EBA	[42]

### 5.2. Drug-Resistant TB

Drug-resistant TB is a growing global health concern that significantly contributes to morbidity and mortality [43]. MDR-TB is one of the most prevalent forms of drug-resistant TB and is commonly the result of genetic mutations in the rpoB and katG genes, which, respectively, confer resistance to RIF and INH [12]. RIF resistance most often results from mutations in the RRDR, with the point mutations S450L, H445D, H445Y, and H445R showing the highest level of RIF-resistance. These mutations disrupt the RIF-binding pocket, causing a loss of hydrogen bonding and, in some cases steric hindrance (S450L and H445Y) preventing RIF from binding [44]. INH resistance is due to mutations in katG, which codes for a catalase peroxidase enzyme, which is required for activation of INH. The most prevalent katG mutation, S315T, induces a conformational change that reduces the binding affinity of the enzyme for INH. As a result, INH cannot be oxidized and remains inactive [45]. A recent study used CRISPR-Cas to detect the S315T mutation at concentrations as low as 1%, demonstrating the increased sensitivity and specificity. Using CRISPR-Cas as a detection method allows for more rapid detection and quicker treatment [46].

While high-dose INH has been explored as a potential strategy against *katG*-mediated resistance, a recent phase 2 trial demonstrated minimal EBA in most patients. Limited efficacy was only observed among slow N-acetyltransferase 2 acetylators, who achieved higher drug concentrations, thus suggesting that high-dose INH is unlikely to be effective in the majority of individuals with *katG* mutations [47]. These findings highlight the necessity of more effective treatment alternatives in patients with drug-resistant TB.

Recent years have seen the introduction of novel drugs such as bedaquiline, pretomanid, and delamanid, alongside optimized use of existing agents like linezolid and later-generation fluoroquinolones [43]. The American Thoracic Society and the Infectious Disease Society of America updated their treatment guidelines in 2024 to recommend a 6-month oral regimen of bedaquiline, pretomanid, and linezolid (BPaL) for RIF-resistant, fluoroquinolone-resistant TB; for cases in which the strain remains susceptible to fluoroquinolones, moxifloxacin is added to form the BPaLM regimen [32]. Multiple clinical trials have supported these shortened regimens [48,49,50]. The TB-PRACTECAL trial found that a 24-week BPaLM regimen was non-inferior to the previous 36- to 80-week standard of care treatment for RIF-resistant TB [48]. Additionally, Paton et al. [49] demonstrated that an 8-week initial intensive regimen with bedaquiline and linezolid, along with INH, PZA, and EMB, was non-inferior to the standard 24-week treatment. Bedaquiline is thought to be suitable for this 8-week initial treatment due to its long half-life and may extend efficacy beyond administration. Patients on the 8-week initial treatment also reported higher motivation to remain adherent to the full course [49]. While shortened treatment regimens are beneficial to improving adherence, they usually require a higher dose or include more drugs [50]. A 2024 randomized controlled trial found that 24 weeks of shortened regimens with either levofloxacin or bedaquiline in combination with linezolid, cycloserine, clofazimine, and PZA were less hepatotoxic than the WHO injectable containing regimen. Additionally, the study found that hepatotoxicity manifests 2–3 months following initiation of RIF-resistant TB treatment, so ongoing liver function testing should be used [50].

There is also research working to optimize drug dosing and safety profiles [51,52]. For example, lower exposure to linezolid by using 600 mg of linezolid for 9–13 weeks followed by 300 mg of linezolid to complete 26 weeks of treatment in BPaL regimens has been shown to maintain efficacy while significantly reducing toxicity [51]. Supporting this, Conradie et al. [52] found that a 600 mg, 26-week regimen of linezolid had the most favorable risk-benefit profile. Compared to the higher dose 1200 mg linezolid group, the 600 mg linezolid group had fewer incidences of peripheral neuropathy and myelosuppression. When compared to the 600 mg, 9-week regimen, the 26-week course resulted in a lower incidence of bacteriologic failure [52]. Pharmacokinetic-pharmacodynamic modeling of linezolid using time-to-positivity (TTP) and colony-forming unit data from a phase 2 trial demonstrated an exposure-dependent bactericidal activity against TB across six orally administered dosing regimens. Higher cumulative drug exposure increased the rate of bactericidal clearance, with 1200 mg once daily showing the optimal response. However, the known toxicity that is associated with higher doses is a concern that necessitates further evaluation. The study also found that the presence of pulmonary cavities was associated with lower baseline TTP, which highlights the impact of disease severity on treatment response [53]. Additionally, pharmacokinetic-pharmacodynamic modeling of INH dosage and bactericidal activity against *inhA*-mutated *M. tuberculosis* demonstrated that increased dosage of INH based on the N-acetyltransferase 2 status of the patient was comparable to the standard 5 mg/kg INH dose for patients with drug-sensitive TB; the dose was increased to 10 mg/kg for slow acetylators and 15 mg/kg for fast acetylators [54].

A recent clinical trial evaluated a four-month regimen consisting of bedaquiline, pretomanid, moxifloxacin, and pyrazinamide (BPaMZ) for the treatment of TB. The regimen demonstrated promising bactericidal activity and showed potential to shorten treatment duration for both drug-susceptible and drug-resistant TB. However, a heightened risk of hepatic adverse events, particularly associated with the combination of pretomanid and PZA, poses a limitation to the use of this regimen in high-burden regions that lack the ability to closely monitor hepatotoxicity [55].

A 2022 randomized controlled trial found that adding clofazimine to MDR-TB treatment significantly improves cure rates [56]. However, clofazimine is also associated with significant QT interval prolongation and is dependent on plasma concentration. Therefore, increased EKG monitoring is recommended for patients receiving a loading dose of clofazimine [57]. Another recent phase 3 clinical trial assessed the safety and effectiveness of five 9-month all-oral regimens with different combinations of bedaquiline, delamanid, linezolid, levofloxacin or moxifloxacin, clofazimine, and PZA for treatment of RIF-resistant, fluoroquinolone-susceptible TB. Three of the five regimens evaluated demonstrated comparable effectiveness to the current standard therapy, indicating promising alternatives for shorter and more tolerable treatment options. Similarly to the aforementioned study, a higher percentage of participants in the experimental groups experienced hepatotoxic events, once again highlighting the importance of careful monitoring of liver enzyme levels during treatment [58]. Table 2 summarizes the various drug-resistant TB regimens.

**Table 2:** Summary of novel and alternative treatment regimens for drug-resistant TB compared to standard 6-month regimens. Regimens include shortened durations, modified dosing strategies, or non-standard drugs currently under investigation in clinical trials or recent studies. Key findings include the primary efficacy outcomes reported in each study relative to the standard regimen.

Regimen	Drugs Used	Treatment Duration	Key Findings	Study/Source
BPaLM Standard Treatment (Reference)	Bedaquiline, Pretomanid, Linezolid, Moxifloxacin	6 months	Baseline Comparison Regimen for Rifampicin-Resistant, Fluoroquinolone-Susceptible TB	[2]
BPaL Standard Treatment (Reference)	Bedaquiline, Pretomanid, Linezolid	6 months	Baseline Comparison Regimen for Rifampicin- and Fluoroquinolone- Resistant TB	[36]
8-Week Intensive Bedaquiline-Linezolid Regimen	Bedaquiline, Linezolid, Isoniazid, Pyrazinamide, Ethambutol	8 weeks (Followed by Continuation Phase)	Non-inferior to standard 24-week treatment; Improved Adherence	[48,49]
Shortened Regimen with Levofloxacin or Bedaquiline Regimen	Linezolid, Cycloserine, Clofazimine, Pyrazinamide, Levofloxacin or Bedaquiline	24 weeks	Less hepatotoxicity than injectable-based regimens	[50]
Lower Exposure to Linezolid Regimen	600 mg Linezolid for 9–13 weeks + 300 mg Linezolid for remaining treatment (as part of BPaL)	26 weeks	Maintains efficacy of standard treatment	[51]
Lower Dose Linezolid Regimen	600 mg of Linezolid (as part of BPaL)	26 weeks	Fewer incidents of peripheral neuropathy, myelosuppression, and bacteriologic failure	[52]
Higher Exposure Linezolid	1200 mg Linezolid with multiple dosing regimens	N/A *	Increased bactericidal clearance	[53]
Increased Isoniazid Dosing (in patients with inhA mutation)	Isoniazid 10 mg/kg (slow acetylators), 15 mg/kg (fast acetylators)	N/A *	Efficacy comparable to standard dose in drug-sensitive TB	[54]
BPaMZ Regimen	Bedaquiline, Pretomanid, Moxifloxacin, and Pyrazinamide	4 months	Strong bactericidal activity; increased risk of hepatic adverse events	[55]
Clofazimine Regimen	Bedaquiline, Levofloxacin, Linezolid, Cycloserine, and 100 mg Clofazimine	6 months	Improved cure rates	[56]
All-oral 9-month Regimens	Various combinations of Bedaquiline, Delamanid, Linezolid, Levofloxacin or Moxifloxacin, Clofazimine, and Pyrazinamide	9 months	Comparable to standard treatment; increased hepatotoxic adverse events	[58]

* Pharmacokinetic-pharmacodynamic modeling.

## 6. Adjunctive Therapies

### 6.1. Metformin

Diabetes mellitus (DM) is a chronic condition that affects more than 400 million people globally [59]. DM is known to increase the risk of developing TB due to impaired immune responses caused by chronic hyperglycemia. Elevated blood glucose levels reduce the number and function of innate immune cells, including macrophages, dendritic cells, and natural killer cells, impairing their ability to recognize, phagocytose, and clear *M. tuberculosis* [60]. Thus, DM is associated with poorer TB treatment outcomes and increased clinical complexity [61,62]. However, metformin, a commonly prescribed first-line treatment for type 2 DM, has been studied in recent years for its possible use as an adjunctive therapy in TB treatment, as it is known to have immunomodulatory and anti-inflammatory effects [63]. A 2021 randomized controlled trial investigated the effect of adding metformin to standard anti-TB treatment for non-diabetic patients with newly diagnosed RIF-susceptible TB and had promising findings that supported the use of metformin as an adjunctive therapy for TB treatment [64]. After 8 weeks of treatment with the metformin dosages of 500 mg oral daily for 1 week and 1000 mg oral daily for the following 7 weeks, the study determined that the addition of metformin did not significantly reduce the time to sputum culture conversion. However, metformin use was associated with quicker resolution of cavities on chest X-ray. It also led to significantly lower circulating plasma levels of the proinflammatory markers IL-17A, IL-1β, TNF-α, and IFN-γ. These findings were indicative of a possible reduction in post-TB sequelae. Of note, however, was the initial increase in mild nausea and vomiting during the first week of metformin use, though gastrointestinal adverse effects are a known side effect of metformin use and the utilization of a loading dose prior to the administration of the full metformin dose has been shown to mitigate these effects [64,65,66,67]. Metformin was also recently studied to assess its effect on RIF, INH, and PZA plasma levels in 60 non-diabetic individuals with drug-sensitive pulmonary TB. The study determined that metformin co-administration significantly increased the clearance of these anti-TB drugs and reduced their plasma exposure compared to the control group receiving standard TB therapy alone. The authors noted that the small sample size was a limitation and recommended larger studies to better understand host drug–drug interactions and pharmacogenetic influences [68].

Population-based observational studies have also explored the clinical impact of metformin in TB-infected individuals with diabetes. A systematic review of twelve observational studies found that metformin use among individuals with diabetes was significantly associated with a reduced risk of developing active TB, lower TB-related mortality, and higher rates of sputum culture conversion at 2 months. However, metformin did not appear to reduce the risk of latent TB infection or TB relapse, suggesting its primary benefit may lie in enhancing host immune response and supporting treatment outcomes in active TB disease [69].

In addition to clinical outcomes, mechanistic studies have explored the immunological and endocrine effects of metformin relevant to TB. The implication of using metformin as an adjunctive therapy for TB treatment was also investigated in a recent study that assessed the effect of metformin on cortisol and dehydroepiandrosterone (DHEA) synthesis in adrenal cells. The study found that DHEA synthesis was enhanced while cortisol homeostasis was maintained, mycobacterial loads in macrophages were reduced, and proinflammatory cytokine expression was modulated, all of which indicated metformin’s potential to promote immunity against TB [70]. Similarly, another clinical study evaluating metformin as an adjunct to standard TB therapy found that metformin significantly reduced circulating levels of proinflammatory chemokines, including CCL1, CCL3, CXCL2, and CXCL10, at both 2 and 6 months of treatment. This effect was especially pronounced in individuals with high bacterial burden or cavitary disease, suggesting that metformin may help regulate excessive inflammation and support improved immunological balance during TB treatment [71].

Furthermore, a human in vivo and in vitro study further supported metformin’s potential as an adjunctive host-directed therapy (HDT) by demonstrating that metformin modulated immune function through downregulation of type I interferon signaling and proinflammatory cytokine production, while enhancing phagocytosis and reactive oxygen species generation. These effects were associated with altered gene expression and immune cell distribution, suggesting that metformin helps promote a more balanced immune response to *M. tuberculosis* by limiting excessive inflammation while supporting bacterial clearance [72]. In addition, a recent phase 2 randomized controlled trial evaluated metformin as well as dovramilast in patients with RIF-resistant TB to assess their safety and preliminary efficacy. Results of the study suggested that these therapies may reduce lung damage and improve treatment outcomes in this patient population [73].

Overall, the findings from these recent studies highlight metformin’s potential to improve clinical outcomes for TB patients as an adjunctive therapy. Further research is necessary to fully understand its mechanisms and to optimize its use in TB treatment strategies. While early studies show promise, most have been small-scale or limited to specific subgroups, making their generalizability uncertain. Specifically, larger clinical trials, well-defined patient selection criteria, and regulatory evaluation will be essential before metformin can be considered for inclusion in TB treatment guidelines.

### 6.2. N-Acetylcysteine and Glutathione

N-Acetylcysteine (NAC) is a temperature stable derivative of cysteine, and during severe or sustained oxidative stress, it replenishes cellular glutathione (GSH), which has antimicrobial, antioxidant, and immunomodulatory properties [74]. GSH can balance free radical levels that have become unbalanced due to TB infection [75].

A nested, randomized controlled trial within the TB SEQUEL cohort study examined the effect of 1200 mg of oral NAC twice daily as an adjunctive treatment along with standard TB treatment. Within the treatment group, GSH levels rose rapidly during the first 14 days of administration and remained elevated until NAC was discontinued. Additionally, FEV1% and FVC% increased in the NAC treatment group compared to the control group, and had effects lasting beyond NAC administration [76]. Analysis of sputum and blood samples found that while NAC administration showed anti-inflammatory effects by decreasing TNF production, it did not show any direct anti-microbial effects. The authors hypothesized that this may be due to insufficient intracellular concentration due to oral administration of NAC. There were no ex vivo effects of NAC or GSH on anti-microbial effects of standard TB drugs [77].

Sukumaran et al. [78] evaluated liver function tests (AST, ALT, ALP, bilirubin) and oxidative stress biomarkers (NO, MDA, GSH) after the administration of 600 mg of NAC along with first-line TB drugs to evaluate hepatoprotective effect of NAC. They found significant reduction ALT and AST from baseline values after 4 weeks of NAC administration. ALT and bilirubin reduction persisted at 8 weeks even after discontinuation of NAC. Oxidative stress biomarkers were similarly reduced and found to persist after stopping NAC [78].

A 2025 systematic review found that NAC and GSH consistently improve lung function, reduce oxidative stress, and, in some studies, accelerate septum conversion. Additionally, the review found that NAC showed a hepatoprotective effect, reducing liver enzyme elevation and incidence of drug-induced hepatotoxicity; these findings support NAC and GSH as promising adjunctive therapies [79]. The integration of NAC and GSH into clinical practice will require further research on dose and delivery method optimization to achieve therapeutic intracellular concentrations and confirming long-term safety.

### 6.3. Other Host Directed Therapies

Several other HDTs are being investigated to improve treatment outcomes in TB, particularly by enhancing immune regulation, reducing lung damage and improving long-term functional recovery [80,81,82,83]. A 2020 double-blind randomized controlled trial assessed the impact of low-dose aspirin (100 mg) in patients with drug-sensitive TB and Type 2 DM. In addition to standard anti-TB therapy and insulin-based diabetes management, aspirin treatment showed improvement in clinical signs, increased rate of sputum conversion, and reduced inflammatory markers and pulmonary cavitary lesions [80].

Looking toward novel immune-modulating compounds, a 2021 study investigated several adjunctive TB HDTs, including CC-11050, a type 4 phosphodiesterase inhibitor, and everolimus, an mTOR inhibitor. They found that both CC-11050 and everolimus enhanced the recovery of FEV1 and recommended further evaluation [81].

Building on the growing research of adjunctive therapies, a phase 2B randomized controlled clinical trial evaluated the effect of 40 mg of atorvastatin as an adjunctive treatment in addition to the intensive phase of standard first line anti-TB drugs. Atorvastatin was found to be associated with higher rates of sputum culture negativity and reduction in mycobacterial burden. Patients in the treatment group were also found to have improved chest X-ray severity scores and most notably reduction in incidence of peripheral neuropathy [82].

Additionally, a 2025 pilot trial suggests that azithromycin as an adjunctive treatment to first-line TB treatment in drug-sensitive TB may reduce pulmonary inflammation and tissue turnover. In the treatment group, where patients were treated with 250 mg oral azithromycin in addition to the standard of care, there was a reduction in IP-10 concentrations in blood, reduced neutrophil percentages in sputum, reduced markers of lung damage and remodeling, including C4M, neutrophil elastase and TFG-Beta. There were no observed effects of azithromycin on the pharmacokinetics of the first line TB treatments [83].

Together, these findings, which are summarized in Table 3, highlight the growing potential of diverse HDTs to complement standard anti-TB regimens by enhancing pathogen clearance, preserving lung structure, and mitigating treatment-related complications, with ongoing research aimed at optimizing their efficacy and integration into TB management [80,81,82,83]. Integration of these host-directed therapies into TB treatment will require larger phase 3 trials to validate their efficacy and safety as well as evaluation of their interactions with standard anti-TB drugs.

**Table 3:** Summary of adjunctive therapies investigated in TB treatment. Therapies include host-directed agents aimed at improving clinical outcomes through immunomodulation, inflammation reduction, or enhanced lung function. Key findings highlight the primary therapeutic effects reported in each study.

Therapy	Key Findings	Study/Source
Metformin	Decreased inflammation, increased immune balance, improved culture conversion, improved radiological outcomes	[64,65,66,67,68,69,70,71,72,73]
N-Acetylcysteine	Increased lung function (FEV1, FVC), reduced TNF, hepatoprotective effects, decreased oxidative stress	[74,75,76,77,78]
Low-Dose Aspirin	Improvement in clinical signs, increased rate of sputum conversion, reduced inflammatory markers and pulmonary cavitary lesions	[80]
CC-11050	Improved FEV1 recovery	[81]
Everolimus	Improved FEV1 recovery	[81]
Atorvastatin	Faster sputum culture conversion, decreased mycobacterial burden, improved chest X-ray scores, reduced peripheral neuropathy	[82]
Azithromycin	Reduced lung inflammation and tissue turnover, decreased IP-10 and neutrophils	[83]

## 7. Implementation Strategies

Ensuring consistent medication adherence is vital for successful treatment of TB and the standard approach has been directly observed therapy (DOT) [36]. Video directly observed therapy (VDOT) has emerged as a more flexible alternative; comparison of VDOT versus the standard in-person DOT showed that there was no statistical differences between the two observed treatment methods. However, VDOT sessions were, on average, much shorter than each DOT session, 16.5 min and 44.1 min, respectively [84]. Additionally, VDOT offers logistical advantages such as avoiding disruptions due to weather or patient travel. In the patient satisfaction survey, patients in both the DOT and VDOT groups felt that observed treatment helped them not miss doses, but patient acceptance of VDOT was higher, VDOT visits were briefer, and patients were more willing to receive VDOT as well as recommend it to other patients [84]. Similarly, Burzynski et al. [85] found that VDOT was non-inferior to in-person DOT. While most patients preferred VDOT, a small percentage of patients declined electronic DOT in favor of in-person DOT. A combination of both VDOT and in-person DOT methods allows a high rate of direct observation; it remains beneficial to implement both strategies to provide coverage across cultures and economic settings [85]. These findings support the use of VDOT as an effective and time-saving alternative to in-person DOT. Implementation of flexible DOT strategies can improve adherence rates across populations while reducing the burden on both patients and healthcare systems [84].

In Uganda, they developed and designed the ZMG’s Active Care and Treatment Strategy (ACTS) Model to provide a bottom-up approach to increase treatment compliance and enhance the awareness of TB. The ACTS model consists of six stages, active ground building, active case finding, active patient compliance, active community supervisions, active treatment management, and active ground assessment, which aim to empower the community and patient. They conducted this study among the low-resource rural communities within three districts in Uganda where the majority of the participants were from lower socio-economic backgrounds and semi-literate. In the first stage of the ACTS model, active ground building, the researchers used digital and non-digital resources to educate participants. The participants that attended the sessions were administered a pre-post questionnaire and found the knowledge levels of how to prevent and treat TB significantly increased (*p* value ≤ 0.01). When they assessed DOT versus VDOT, they found a significant increase in medication compliance (*p* value ≤ 0.05) with participants preferring VDOT due to saving money and time and the app providing them with alerts and information regarding nutrition while receiving treatment [86].

## 8. Limitations

The literature search for this review was restricted to the PubMed database, which may have limited the comprehensiveness of the included studies and excluded relevant articles indexed in other databases. PubMed was chosen for its focus on peer-reviewed biomedical literature. Future reviews should consider including multiple databases to ensure broader coverage of relevant studies.

## 9. Conclusions

This review set out to synthesize recent advances in the management of drug-sensitive and drug-resistant TB, with a focus on novel pharmacologic regimens and host-directed therapies. Collectively, the evidence demonstrates progress towards shorter and more tolerable regimens including higher dose PZA with higher culture conversion rates [38], faropenem substitution regimen with fewer side effects [40], and the BPaMZ regimen with strong bactericidal activity [55]. However, these advancements remain constrained by persistent challenges including drug-toxicity, emerging resistance to new agents, and limited capacity for consistent toxicity monitoring in high-burden settings [16,58]. Adjunctive and host-directed therapies such as metformin, NAC, glutathione, and statins show promise in enhancing host immune responses and mitigating long-term lung damage [64,77,80,81,82,83]. Their integration into clinical practice; however, requires larger trials, standardized dosing strategies, and evaluation for potential drug-interactions.

Advances in diagnostics, particularly WGS and CRISPR-based methods, offer the potential for rapid resistance detection and directed therapies. Continued research is needed to determine how to treat various point mutations that lead to TB resistance and limit off-target effects. Additionally, difficulties may lie with scalability, and cost-effectiveness in resource-limited regions [22,25,26]. Similarly, vaccine candidates beyond BCG represent a promising opportunity for prevention, but still remain in the early phases of clinical development [28]. Implementation strategies such as VDOT and community-based frameworks like the ACTS model underscore the value of patient-centered care [85,86].

All of these advances highlight that meaningful progress against TB will depend on combining shorter, safer drug regimens with host-directed therapies, rapid diagnostics, effective vaccines, and patient-centered implementation strategies to close the gap between research and real-world impact.

## 10. Future Perspectives

The future of TB management hinges on bridging research and clinical practices. As knowledge continues to grow, therapeutics are likely to become more tailed to the specific stage of the disease. In early drug-sensitive TB, optimized regimens such as higher-dose pyrazinamide or faropenem substitution could shorten treatment duration and improve tolerability. For drug-resistant cases, regimens like BPaLM and BPaMZ offer shorter, safer alternatives, while host-directed therapies may help limit lung damage and long-term sequelae. Additionally, research into drug safety and toxicity is critical to ensure that novel regimens are both effective and well-tolerated across diverse patient populations. Future research studies can thus focus on evaluating stage-specific strategies, optimizing dosing, evaluating safety and toxicity, and assessing long-term outcomes to guide personalized TB therapy.

## Data Availability

No new data were created or analyzed in this study. Data sharing is not applicable to this article.

## Abbreviations

ACTS	Active Care and Treatment Strategy
BCG	Bacille Calmette-Guérin
BPaL	Bedaquiline, Pretomanid, and Linezolid
BPaLM	Bedaquiline, Pretomanid, Linezolid, and Moxifloxacin
CARP	Cas9/gRNA-assisted quantitative Real-Time PCR
CAMPER	Cas9 and Mutagenic Polymerase for Evolving Resistance
CRISPR-CAS	Clustered Regularly Interspaced Short Palindromic Repeats-CRISPR Associated
DHEA	Dehydroepiandrosterone
DM	Diabetes Mellitus
DOT	Directly Observed Therapy
DST	Drug Susceptibility Test
EBA	Early Bactericidal Activity
EMB	Ethambutol
ESAT-6	Early Secreted Antigenic Target 6
HDT	Host-Directed Therapy
INH	Isoniazid
MDR-TB	Multi-Drug-Resistant Tuberculosis
NAC	N-Acetylcysteine
PZA	Pyrazinamide
RIF	Rifampicin
RRDR	RIF resistance-determining region
TB	Tuberculosis
TTP	Time-to-Positivity
VDOT	Video Directly Observed Therapy
WGS	Whole Genome Sequencing
WHO	World Health Organization
